# γ-Tubulin in microtubule nucleation and beyond

**DOI:** 10.3389/fcell.2022.880761

**Published:** 2022-09-01

**Authors:** Vadym Sulimenko, Eduarda Dráberová, Pavel Dráber

**Affiliations:** Laboratory of Biology of Cytoskeleton, Institute of Molecular Genetics of the Czech Academy of Sciences, Prague, Czechia

**Keywords:** microtubule nucleation, αβ-tubulin dimer, γ-tubulin functions, γ-tubulin isotypes, γ-tubulin ring complexes (γ-TuRC)

## Abstract

Microtubules composed of αβ-tubulin dimers are dynamic cytoskeletal polymers that play key roles in essential cellular processes such as cell division, organelle positioning, intracellular transport, and cell migration. γ-Tubulin is a highly conserved member of the tubulin family that is required for microtubule nucleation. γ-Tubulin, together with its associated proteins, forms the γ-tubulin ring complex (γ-TuRC), that templates microtubules. Here we review recent advances in the structure of γ-TuRC, its activation, and centrosomal recruitment. This provides new mechanistic insights into the molecular mechanism of microtubule nucleation. Accumulating data suggest that γ-tubulin also has other, less well understood functions. We discuss emerging evidence that γ-tubulin can form oligomers and filaments, has specific nuclear functions, and might be involved in centrosomal cross-talk between microtubules and microfilaments.

## Introduction

The microtubule cytoskeleton is essential for vital cellular functions such as cell division, maintenance of cell shape, organelle positioning, intracellular transport, and cell migration. Microtubules are dynamic in nature and oscillate stochastically between phases of assembly and disassembly in a process known as “dynamic instability of microtubules” (Mitchison and Kirschner, 1984). The major building components of microtubules are αβ-tubulin heterodimers that form cylinders with a diameter of ∼25 nm. αβ-Tubulins are linked head-to-tail and form a polar protofilament. The lateral connection of thirteen protofilaments forms a left-handed helical microtubule wall. Polar microtubules have structurally distinct ends: a fast-growing plus end (+) that exposes β-tubulin and a slow-growing minus end (-) that exposes α-tubulin. Both subunits bind GTP, but hydrolysis occurs only at the β-subunit (Nogales and Wang, 2006). In cells, the (-)-ends of microtubules are anchored in microtubule organization centers (MTOCs), whereas the unanchored (+)-ends are very dynamic. Due to the dynamic properties, the microtubule network remodels in response to various signaling stimuli.

The low concentration of αβ-tubulin dimer in the cytosol prevents spontaneous nucleation of microtubules. Therefore, nucleation occurs from MTOCs. The centrosome, which consists of two centrioles surrounded by pericentriolar material (PCM), is the major MTOC in mammalian cells. In addition, the centrosome locally concentrates various signaling molecules, including kinases and phosphatases, integrates various signaling pathways (Arquint et al., 2014) and is involved in actin filament organization (Farina et al., 2016; Inoue et al., 2019). Microtubules are also nucleated from other MTOCs such as the Golgi apparatus, pre-existing microtubules, nuclear envelope, chromatin, cell cortex endosomes and mitochondria as reviewed recently (Paz and Lüders, 2018; Akhmanova and Kapitein, 2022). These noncentrosomal MTOCs play important roles in the construction and regulation of the dynamic microtubule system.

γ-Tubulin (Oakley and Oakley, 1989) is a highly conserved member of the tubulin family (Ludueña, 2013), present at less than 1% the level of αβ-tubulin (Stearns et al., 1991). It combines with other proteins to form γ-tubulin complexes, which are the basic elements for nucleation of microtubules from MTOCs at various cellular sites as reviewed previously (Oakley et al., 2015; Petry and Vale, 2015; Tovey et al., 2018; Thawani and Petry, 2021).

This review focuses on recent research and emerging issues related to the γ-tubulin functions. Particular attention is paid to the structure of the γ-tubulin ring complex (γ-TuRC), the regulation of centrosomal microtubule nucleation, the ability of γ-tubulin to form oligomers, and the nuclear functions of γ-tubulin. We also discuss the role of γ-TuRC in centrosomal microfilament/microtubule cross-talk.

### γ-Tubulin isotypes and posttranslational modifications

Isotypes of α- and β-tubulins, encoded by multiple genes, differ mainly in their C-terminal tails (CTTs). The differences between isotypes are often evolutionarily highly conserved, indicating their functional importance (Ludueña, 1993). Nine isotypes for each tubulin subunit have been identified in humans. Some isotypes are ubiquitous, while others are found only in specialized microtubule assemblies (Ludueña, 2013; Roll-Mecak, 2020). In contrast, in humans, there are only two γ-tubulin genes (*TUBG1* and *TUBG2*) with 94% sequence similarity, which are located in tandem at the 17th chromosome (Wise et al., 2000). The difference between human γ-tubulin-1 and γ-tubulin-2 is only ten amino acids, nine of which are located in the C-terminal domains of the molecules (aa 389–451). Nevertheless, they can be distinguished based on their electrophoretic and immunochemical properties (Ohashi et al., 2016; Dráberová et al., 2017). Both γ-tubulins are capable of nucleating microtubules (Vinopal et al., 2012). While γ-tubulin-1 is ubiquitously found, γ-tubulin-2 is mainly expressed in the brain (Wise et al., 2000; Yuba-Kubo et al., 2005). The function of γ-tubulin-2 is unclear, but based on its accumulation in neuroblastoma cells under oxidative stress and in mature neurons, it may have a prosurvive function. In mature neurons, dominant γ-tubulin-1 may ensure noncentrosomal microtubule nucleation (Dráberová et al., 2017).

The atomic structure of γ-tubulin shows a conformation similar to α- and β-tubulins (Aldaz et al., 2005; Rice et al., 2008). When the defined microtubule polarity is extended to the ends of the αβ-tubulin dimer and each tubulin monomer, the (+)-end of γ-tubulin contacts the (-)-end of α-tubulin. γ-Tubulin shares high homology with β-tubulin in the (+)-end face involved in longitudinal contacts between αβ-tubulin dimers (Inclán and Nogales, 2001). Similar to αβ-tubulin dimers, γ-tubulin binds GTP, which enhances its interaction with αβ-tubulin dimers in both budding yeast *Saccharomyces cerevisiae* (Gombos et al., 2013) and reconstituted human γ-TuRC (Wieczorek et al., 2021).

**FIGURE 1:**
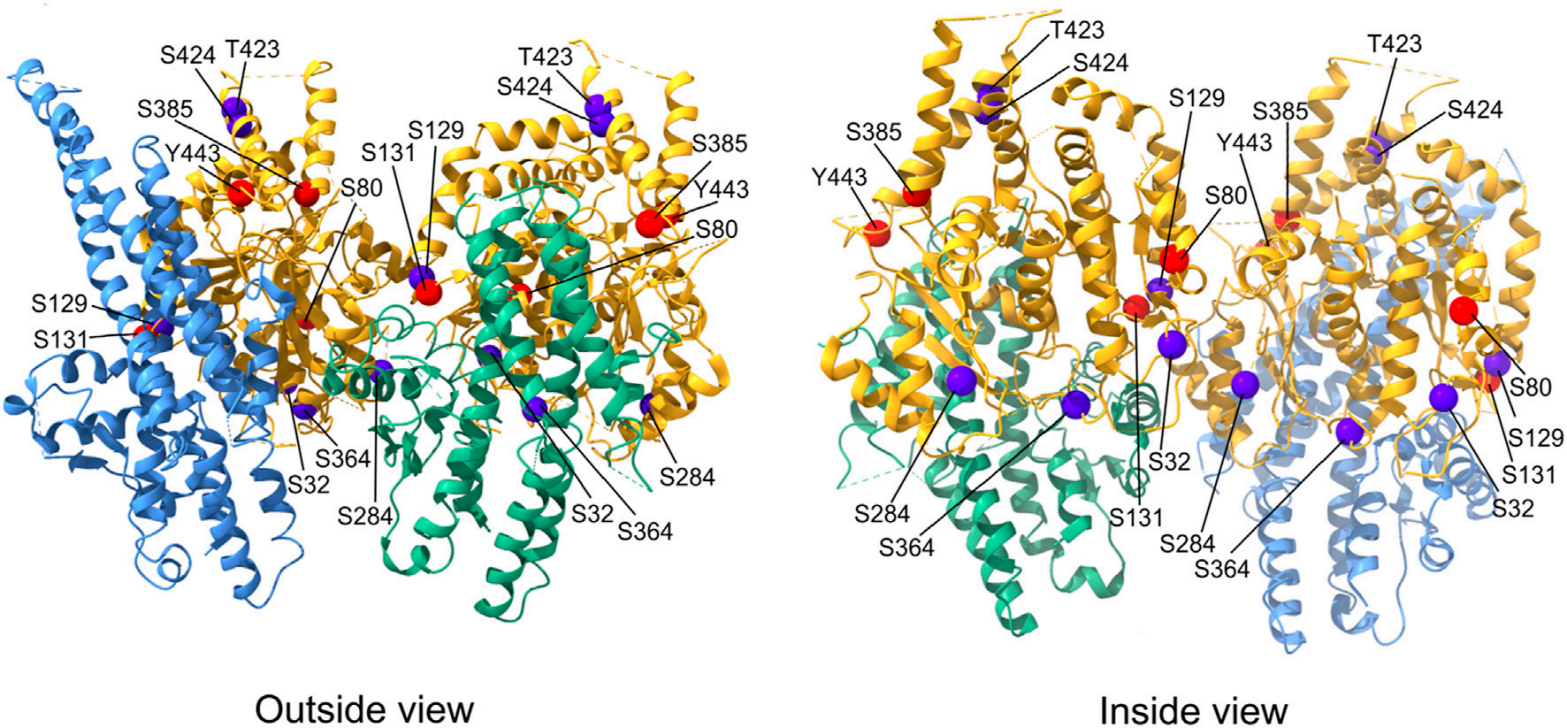
Distribution of phosphorylation sites on human γ-tubulin. Exterior and interior views of the γ-TuSC portion containing γ-tubulins (yellow) interacting with the C-terminal GRIP2 domains of GCP2 (green) and GCP3 (light blue). The position of γ-tubulin phosphorylation sites for which kinases are known are marked with red spheres, phosphorylation sites without corresponding kinases are marked with dark blue spheres. The molecular structure representation is based on native human γ-TuRC (PDB: 6v6s) and was generated using ChimeraX 1.3 software.

Extensive posttranslational modifications (PTMs) of α- and β-tubulin isotypes (Janke and Magiera, 2020) generate multiple charge variants of both subunits, termed tubulin isoforms, which can be separated by isoelectric focusing (Wolff et al., 1982; Linhartová et al., 1992). PTMs of γ-tubulins also generate multiple charge variants that have been distinguished using 2D-PAGE in various systems, including budding yeast (Vogel et al., 2001), nucleated erythrocytes (Linhartová et al., 2002), brains (Détraves et al., 1997; Sulimenko et al., 2002), and various cell lines (Kukharskyy et al., 2004; Dráberová et al., 2017). Of the PTMs of γ-tubulin, most data have been collected on its phosphorylation. Large-scale phosphoproteomic analysis of spindle pole bodies (SPBs) in budding yeast revealed multiple phosphorylation sites on γ-tubulin (Tub4) (Keck et al., 2011; Lin et al., 2011; Fong et al., 2018). Phosphomimetic mutations of highly conserved Tub4 sites resulted in spindle assembly defects (S360) (Keck et al., 2011; Lin et al., 2011), increased number of SPB microtubules (Y445) (Vogel et al., 2001), defects in spindle alignment (Y362) (Shulist et al., 2017)**,** induced metaphase arrest (S74 and S100) (Lin et al., 2011), and cell cycle delay (S71) (Fong et al., 2018). Overall, these data strongly suggest that phosphorylation of γ-tubulin is important for the control of microtubule organization in the course of cell cycle in yeast. Multiple phosphorylation sites on γ-tubulin are also important for basal body assembly and stability, as shown in the ciliate *Tetrahymena thermophila* (Joachimiak et al., 2018). Phosphorylation analysis of human mitotic protein complexes revealed multiple phosphorylation sites on γ-tubulin (Hegemann et al., 2011), but the corresponding kinases are largely unknown. In mammals, the kinase BRSK1 (SADB), which controls cell cycle progression, phosphorylates γ-tubulin at S131 and S385. Phosphorylation at the S131 residue controled centrosome duplication (Alvarado-Kristensson et al., 2009), while phosphorylation at the S385 residue regulated cellular localization of γ-tubulin. Phosphomimetic S385D γ-tubulin translocated to the nucleus and influenced the execution of S phase (Eklund et al., 2014). Recently, the nonreceptor tyrosine kinase c-Abl was reported to phosphorylate γ-tubulin at Y443, the equivalent residue of Y445 in yeast γ-tubulin. Phosphorylation at the Y443 residue promoted assembly of γ-TuRC and nucleation of centrosomal microtubules (Wang et al., 2022). γ-Tubulin may also be a substrate for Cdk2 (cyclin-dependent kinase 2) at S80 (Chi et al., 2008). Additional serine and threonine phosphorylation sites (S32, S129, S284, S364, T423, and S424) have been identified by mass spectrometry on human γ-tubulin (PhosphoSitePlus database), but their functional significance is unknown. The distribution of known phosphorylation sites on the human γ-tubulin molecule is shown in Figure 1.

Ubiquitination is another PTM relevant to γ-tubulin. Monoubiquitination of γ-tubulin by the BRCA1 (breast cancer type 1 susceptibility protein)/BARD1 (BRCA1-associated RING domain protein 1) E3 ligase complex results in detachment of γ-tubulin from the centrosome and inhibition of microtubule nucleation (Hsu et al., 2001; Starita et al., 2004; Sankaran et al., 2005). On the other hand, removal of ubiquitin from γ-tubulin by the deubiquitylase BAP1 (BRCA1-associated protein-1) leads to accumulation of unmodified γ-tubulin at the centrosome (Zarrizi et al., 2014). Polyubiquitination of γ-tubulin by the E3 ligases cullin 1, cullin 4A, and cullin 4B, followed by its proteosomal degradation, plays an important role in the dismantling of γ-tubulin complexes (Thirunavukarasou et al., 2015; Yin et al., 2021). Finally, acetylation of human γ-tubulin (K397, K400) was also identified by mass spectrometry (PhosphoSitePlus database), but the function is unknown.

### γ-Tubulin nucleation complexes

γ-Tubulin together with its associated proteins forms complexes that are essential for microtubule nucleation. A large fraction of cytosolic γ-tubulin exists in a tetrameric complex with γ-tubulin complex protein (GCP)2 and GCP3 in stoichiometry 2:1:1, termed the γ-tubulin small complex (γ-TuSC), with a molecular weight of ∼300 kDa (Oegema et al., 1999; Kollman et al., 2008). In budding yeast, where Spc97 and Spc98 are homologs of GCP2 and GCP3, respectively, the γ-TuSC represents a major structural unit of the γ-TuRC (Kollman et al., 2015). In higher eukaryotes, γ-TuSCs with additional γ-tubulins and GCP4-6 form the helical ring of γ-TuRC with a molecular weight of ∼2.2-MDa. γ-TuRC provides a template that mimics the geometry of microtubules and stimulates microtubule nucleation (Moritz et al., 1995; Zheng et al., 1995; Kollman et al., 2010).

GCP2-6 each bind directly to γ-tubulin to form GCP-γ-tubulin heterodimers (called spokes). Spokes assemble into a left-handed, cone-shaped structure that controls microtubule assembly and facilitates lateral interactions between αβ-tubulin dimers (Kollman et al., 2011). Two short homologous regions are unique to GCPs: the N-terminal GRIP (γ-tubulin ring protein) 1 domain and the C-terminal GRIP2 domain. The flexible connection between these domains allows rearrangement of the γ-tubulin positions in the complex. The GRIP2 domains interact with γ-tubulins, while the GRIP1 domains form the primary interface between GCP proteins (Gunawardane et al., 2000; Kollman et al., 2011). Detailed γ-TuRC structures have recently been uncovered by four independent studies that provide mechanistic insights into how microtubules are templated from γ-TuRC (Wieczorek et al., 2020b; Consolati et al., 2020; Liu et al., 2020; Zimmermann et al., 2020). Cryo-EM reconstructions showed that γ-TuRC has a width of ∼30 nm and a height of ∼25 nm. The 14 spokes are aligned laterally to form a short helix, and the γ-tubulins are located on the open side of the cone, in the C-terminal region of each GCP. Spoke positions 1 and 14 partially overlap. Spoke positions 1-8 are occupied by four γ-TuSCs, whereas spoke positions 9–14 contain GCP4, GCP5, GCP4, and GCP6, and a terminal γ-TuSC. All studies identified a scaffold in the complex interior, called the lumenal bridge, which surprisingly also contains actin. In addition to actin, the luminal bridge includes two small molecules of MZT1 (mitotic spindle organizing protein 1), the N-terminus of GCP6 and the N-terminus of GCP3 (Wieczorek et al., 2020a).

**FIGURE 2:**
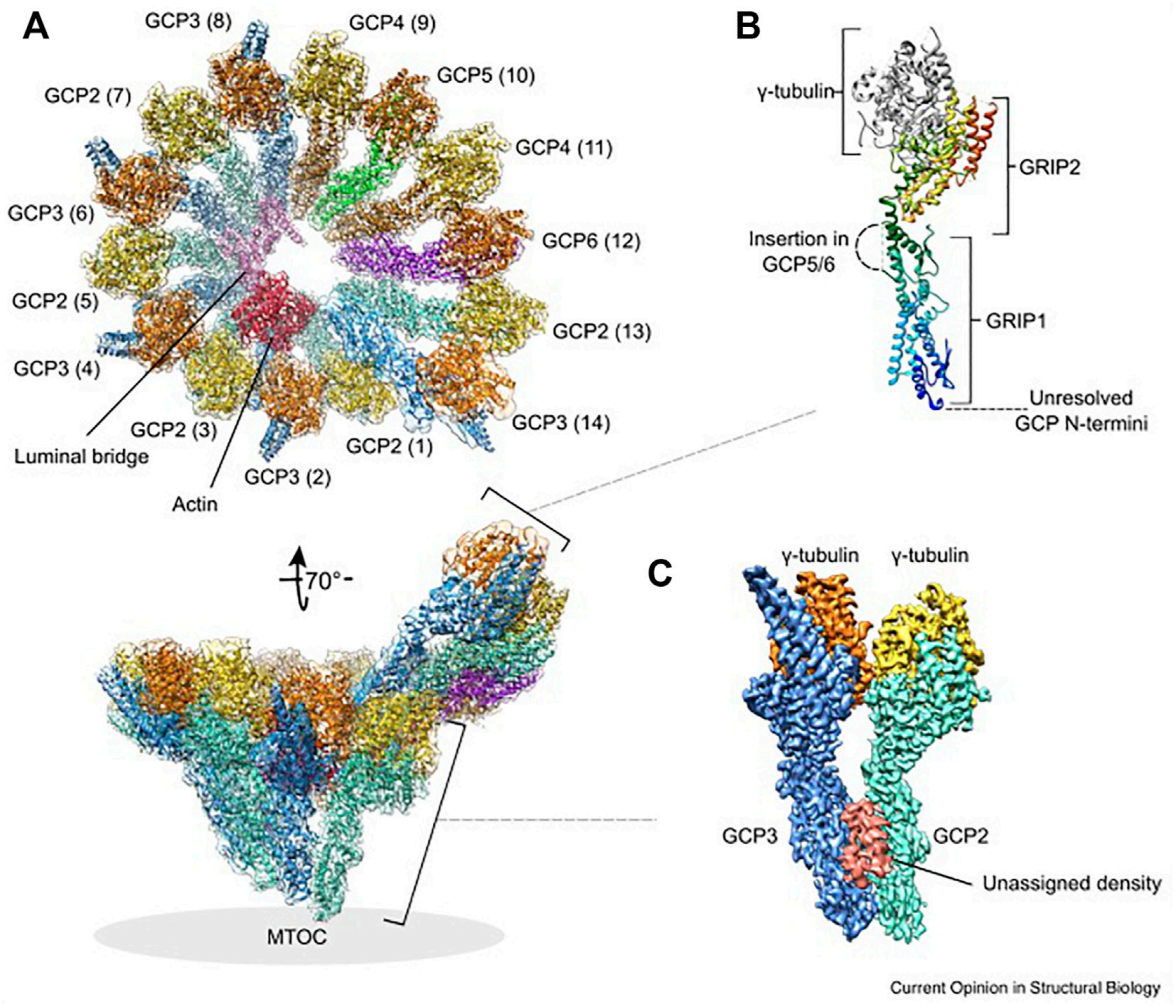
Structure and molecular architecture of human γ-TuRC. **(A)** General architecture of the left-handed γ-TuRC spiral as determined by cryo-EM single-particle analysis, resolution 3.8 Å. γ-Tubulins (yellow, orange), GCP2 (aquamarine), GCP3 (blue), GCP4 (brown), GCP5 (green), GCP6 (purple), actin (red) and the luminal bridge (pink) are shown. The spokes (GCP-γ-tubulin heterodimers) are numbered (1–14 in brackets). In the tilted view, the approximate location of the MTOC is indicated. The orientation of subcomplexes shown in panel **(B)** and **(C)** is indicated. **(B)** General architecture of a GCP-γ-tubulin spoke. The GCP N-terminal GRIP1 and C-terminal GRIP2 domains are annotated. Unresolved GCP segments are indicated by dashed lines. GCP is shown in rainbow colors from N-terminus (blue) to the C-terminus (red). **(C)** Location of the unassigned density segment (red) present on each GCP(2–3) subcomplex of the human γ-TuRC. This figure was prepared using PDB 6V6S and EMD-21074. Reprinted by permission from Current Opinion in Structural Biology (Zupa et al., 2021).

The assembly of γ-TuRC is modular, starting with the formation of a stable subcomplex of six spokes, consisting of GCP2-3-4-5-4-6, which then expands with the addition of four preformed GCP2-3 units (γ-TuSC), MZT1, and actin (Würtz et al., 2022). DNAseI binds directly to actin with high affinity. The *in vitro* nucleation activity of isolated endogenous γ-TuRC was markedly inhibited after treatment with DNAseI, and saturation of DNAseI with actin abolished this inhibition, suggesting a functional importance of actin in the complex (Liu et al., 2020). Actin has been shown not to be required for assembly of γ-TuRC, but to determine the geometry of the complex and ensure effective nucleation of microtubules (Würtz et al., 2022). On the outer surface of reconstituted γ-TuRC, MZT1, and MZT2 were identified to bind to the N-terminal domains of GCPs. These MZT1/2 proteins may aid in the recruitment of γ-TuRC to the centrosome (Wieczorek et al., 2020a; Consolati et al., 2020; Würtz et al., 2022). The location of γ-tubulin molecules at the interface between γ-TuRC and αβ-tubulin dimers does not correspond exactly to the geometry of microtubules. While spokes 1–8 with four GCP2-3 units (γ-TuSC) follow microtubule symmetry and adopt a “closed conformation,” spokes 9–14 are less tightly aligned and do not serve as a perfect template for microtubule nucleation. They are asymmetric in both diameter and spacing and have an “open conformation” (Wieczorek et al., 2020b; Consolati et al., 2020; Liu et al., 2020; Zimmermann et al., 2020). This could explain why the cytosolic γ-TuRC exhibits low nucleation activity (Consolati et al., 2020). The molecular architecture and structure of γ-TuRC is shown in Figure 2.

The deciphered structure of γ-TuRC supports a model of microtubule nucleation in which γ-tubulins recruit αβ-tubulin dimers and promote their lateral interactions during the early stages of microtubule assembly (Zheng et al., 1995; Keating and Borisy, 2000; Moritz et al., 2000; Wiese and Zheng, 2000). It has been shown that the association of as few as four αβ-tubulin dimers (minimal nucleus) in the rate-limiting step is sufficient for γ-TuRC-mediated nucleation (Consolati et al., 2020; Thawani et al., 2020). This process is thus more efficient than spontaneous nucleation of microtubules in solution, which requires cooperative assembly of eight αβ-tubulin dimers in the rate-limiting step (Thawani et al., 2020). It is supposed that a conformational changes leading to fully closed γ-TuRC, consistent with 13-fold microtubule symmetry, are required to increase the efficiency of γ-TuRC nucleation.

### γ-TuRC activation

Although the mechanisms of γ-TuRC activation are not well understood, there is evidence that activation of γ-TuRC may occur by multiple mechanisms. Activating protein factors, phosphorylation of γ-TuRC-building and activating proteins, or conformational changes after binding of αβ-tubulin could be involved in context-specific activation.

Several candidates might play a role as γ-TuRC activating factors. CDK5RAP2 (cyclin-dependent kinase 5 regulatory subunit-associated protein 2/centrosomal protein 215/Cep215) is the best characterized mammalian activator (Fong et al., 2008). It contains an activating ∼5.5-kDa domain (γ-TuNA/γ-TuRC-mediated nucleation activator 1/centrosomin motif 1/CM1) that is conserved in all eukaryotes among proteins that recruit γ-TuRCs to MTOCs (Lin et al., 2015). *In vitro* experiments with purified γ-TuRCs showed differential effects of CM1 on nucleation activity. When the CM1 domain was added to human γ-TuRC, nucleation activity increased 7.1-fold (Choi et al., 2010). However, when the CM1 domain was added to *Xenopus* γ-TuRC, the activity increased only 1.7-fold (Liu et al., 2020) or only insignificantly (Thawani et al., 2020). On the other hand, functional complexes resembling γ-TuRC were formed when the CDK5RAP2 homolog Mto 1/2 from the fission yeast *Schizosaccharomyces pombe* was added to γ-TuSC (Leong et al., 2019). It has also been shown that binding of the CM1 domain from the budding yeast Spc110 protein to γ-TuSC results in structural changes that facilitate assembly of γ-TuRC (Brilot et al., 2021). It has been suggested that the kinase NME7 (nucleoside diphosphate kinase 7), which copurifies with γ-TuRC (Wieczorek et al., 2020b; Liu et al., 2020), may also serve as an activating factor (Liu et al., 2014). However, when NME7 was added to γ-TuRC nucleation assays, the nucleation activity increased only 2.5-fold (Liu et al., 2014) or insignificantly (Thawani et al., 2020). Since the corresponding substrate of NME7 on γ-TuRC is unknown, the question remains whether NME7 can actually activate γ-TuRC. TPX2 (targeting protein for Xklp2), the multifunctional Ran-GTP-regulated factor for spindle assembly (Roostalu and Surrey, 2017; Tovey and Conduit, 2018), could also serve as an activating protein. High concentrations of human TPX2 stimulated γ-TuRC-dependend microtubule nucleation (Consolati et al., 2020). In contrast, such stimulation was not observed in *Xenopus* (Thawani et al., 2020). These differences may reflect the species-specific activity of TPX2. Recently, the well-characterized microtubule polymerase XMAP215 (*Xenopus* microtubule assembly protein 215 kDa; mammalian ch-TOG [colonic and hepatic tumor overexpressed gene protein]) was shown to interact with γ-tubulin complexes (Gunzelmann et al., 2018; Thawani et al., 2018). It also increases the nucleation activity of γ-TuRC up to 25-fold (Consolati et al., 2020; Thawani et al., 2020). It has been proposed that XMAP215 complements γ-TuRC dependent nucleation. XMAP215 first associates with the γ-TuRC and then delivers αβ-tubulin interacting with its TOG domains to the γ-TuRC and subsequently to the growing microtubule end (Thawani et al., 2020). The open question is whether the activity of XMAP-215 is synergistic or additive with γ-tubulin (King et al., 2020).

As described in the previous text, γ-tubulin has multiple phosphorylation sites, and its phosphorylation can modulate the conformational changes required for γTuRC activation. Phosphorylation of sites at the (+) end of γ-tubulin could directly regulate interactions with αβ-tubulin dimers, and the same is true for phosphorylation of sites at the lateral contacts between γ-tubulin and αβ-tubulin dimers (Kollman et al., 2015). Some of the known phosphorylation sites on the human γ-tubulin molecule are located at important interface between γ-TuSCs and may affect the formation of γ-TuRC (Figure 1).

GCPs are also phosphorylated (Hegemann et al., 2011; Santamaria et al., 2011; Fong et al., 2018; Brilot et al., 2021). Surprisingly, phosphorylation at most of the mapped sites on γ-TuSC appears to destabilize the assembled γ-TuRC. On the other hand, a stabilizing effect of phosphorylation was predicted at two sites (Brilot et al., 2021). This highlights that phosphorylation and dephosphorylation may play complex modulatory roles in the activation of γ-TuRC. In higher eukaryotes, only a few kinases are known to phosphorylate GCPs. GCP6 is phosphorylated by the kinases PLK4 (polo-like kinase 4) (Bahtz et al., 2012) and Cdk1 (cyclin-dependent kinase 1) (Oriolo et al., 2007). Phosphorylation of GCP5 by GSK3β (glycogen synthase kinase 3β) inhibits the accumulation of γ-TuRC at centrosomes (Horio and Oakley, 1994). In addition, activating factors can also be regulated by phosphorylation. It has been reported that binding of human CDK5RAP2 (Hanafusa et al., 2015) or SPD-5 (spindle-defective protein 5), a CM1 domain-containing protein from *Caenorhabditis elegans* (Ohta et al., 2021) to γ-TuRCs depends on phosphorylation. Furthermore, binding of CDK5RAP2 to γ-TuRC is regulated by autoinhibition, and phosphorylation helps to abrogate this activity (Tovey et al., 2021). The activating role of NME7 kinase can be affected by its autophosphorylation (Liu et al., 2014). TPX2 is the major cofactor for the mitotic kinase Aurora A, which is indirectly involved in the regulation of γ-TuRC-driven microtubule nucleation (Kufer et al., 2002; Meunier and Vernos, 2016; Joukov and De Nicolo, 2018). Overall, phosphorylation of building components of γ-TuRC may affect the complex stability. Further regulation of microtubule nucleation activity may occur through phosphorylation of activating proteins.

**FIGURE 3:**
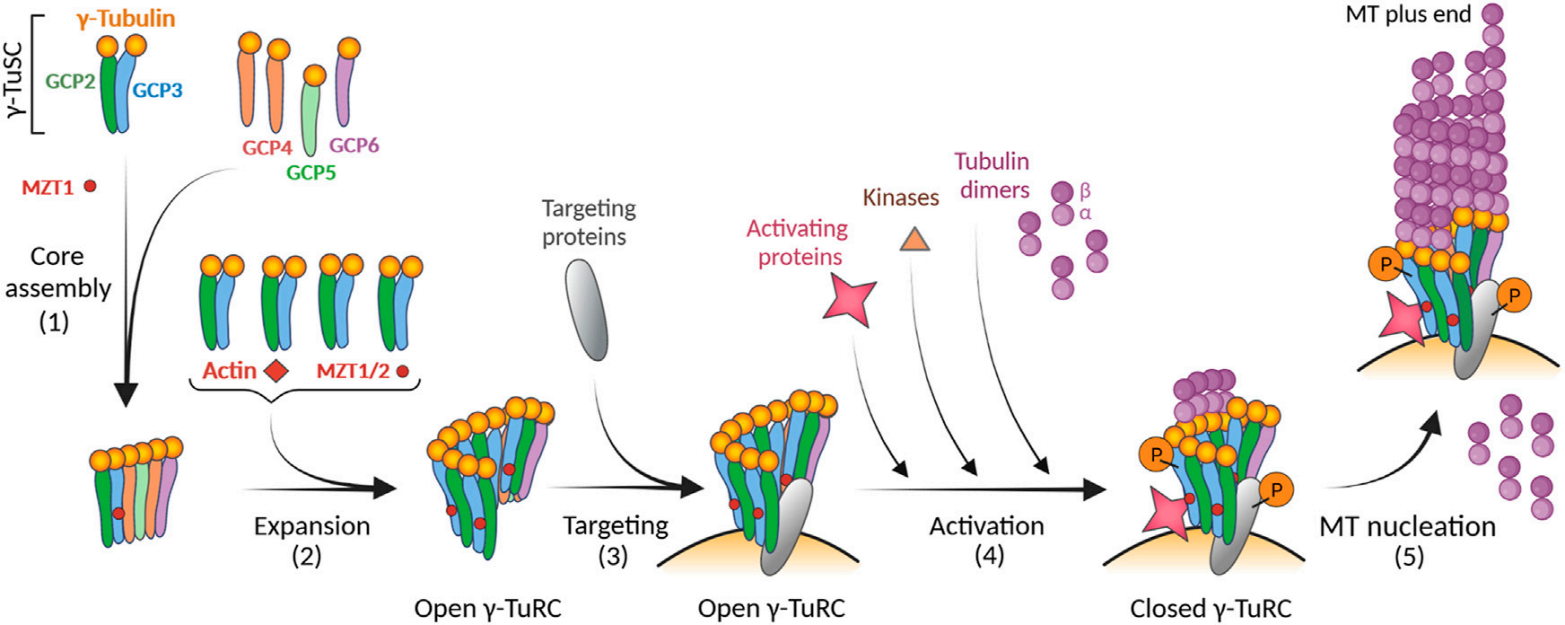
Model for the modular assembly of γ-TuRC and its activation. The first step in the formation of γ-TuRC is the core assembly of the stable subcomplex from γ-TuSC (2 molecules of γ-tubulin and one copy each of GCP2 and GCP3), GCP-γ-tubulin heterodimers (spokes; one molecule of γ-tubulin and one copy each of GCP4, GCP5 or GCP6), and MZT1 **(1)**. During the expansion phase, four additional γ-TuSC units, MZT1/2, and actin are added **(2)**. The resulting γ-TuRCs with the open conformation are concentrated onto centrosomes *via* targeting proteins (e.g., CDK5RAP2, NEDD1) **(3)**. The pitch and diameter of open γ-TuRC are incompatible with those of assembled microtubules. This suggests that the complex undergoes a conformational change through its activation to reduce its diameter before microtubule nucleation. Different modes of activation, including direct binding of activating proteins (e.g., CDK5RAP2, NME7), phosphorylation of γ-TuRC by kinases, or increased concentration of αβ-tubulins, can result in a conformational change leading to a closed γ-TuRC **(4)**. Different types of activation may occur simultaneously. Nucleation-competent γ-TuRC with a closed conformation can then effectively nucleate microtubule (MT) **(5)**. Created with BioRender.com.

Finally, it was suggested that the driving force for achieving a fully closed γ-TuRC conformation might be the arrangement of αβ-tubulin dimers at the γ-TuRC itself. Using computational modelling, it was shown that four laterally associated tubulin dimers at the γ-TuRC create a transition state that closes the γ-TuRC (Thawani et al., 2020). Interestingly, *Caenorhabditis* mitotic centrosomes concentrate soluble αβ-tubulin more than 10-fold compared to the cytoplasm (Baumgart et al., 2019). Thus, the concentration of αβ-tubulin dimers could also modulate the γ-TuRC nucleation activity. An open question is whether, in cells expressing different isotypes of α- and β-tubulins, some tubulin isotypes might be better substrates for microtubule nucleation driven by γ-TuRC (Ti et al., 2018). The model for the modular assembly and activation of γ-TuRC is shown in Figure 3.

### γ-TuRC recruitment to centrosome

In addition to activating proteins, there are other proteins, referred to here as targeting/anchoring proteins, that are involved in the regulation of γ-TuRC-driven microtubule nucleation. They are not essential for the assembly of γ-TuRC, but they help in the recruitment and tethering of the complex to MTOCs. Below, we provide an overview of regulatory proteins important for centrosomal microtubule nucleation in mammalian cells. For a detailed discussion of noncentrosomal microtubule nucleation, we refer reader to recent reviews (Valenzuela et al., 2020; Wilkes and Moore, 2020; Akhmanova and Kapitein, 2022).

Targeting/anchoring of γ-TuRCs to interphase centrosomes is mediated by CDK5RAP2 *via* its CM2 domain (centrosomin motif 2) (Wang et al., 2010) and by NEDD1 (neural precursor cell expressed developmentally down-regulated protein 1/GCP-WD) (Haren et al., 2006; Lüders et al., 2006). Ninein anchors γ-TuRCs to subdistal appendages of the mother centriole (Bouckson-Castaing et al., 1996; Delgehyr et al., 2005). This process may involve dynein complex, which can be activated by ninein (Redwine et al., 2017). γ-TuRC may also be bound to the central region of the mother centriole *via* the centrosomal protein FSD1 (Tu et al., 2018) or to the proximal/PCM region *via* the protein complex MSD1-WDR8 (Hori et al., 2015). Augmin (HAUS) complex interacts with γ-TuRC and is required for branching microtubule nucleation (Goshima et al., 2008). Recently, augmin-γ-TuRC was identified in the lumen of the centriole, and it was shown that γ-TuRCs are recruited to the luminal region by the interaction of augmin with the centriole inner scaffold protein POC5 (Schweizer et al., 2021). Cep192 (centrosomal protein 192), which is implicated in the recruitment of γ-TuRC to centrosome (Gomez-Ferreria et al., 2007; O'Rourke et al., 2014), anchors γ-TuRC both to PCM and to the outer sides of centrioles (Schweizer et al., 2021). Additional proteins such as AKAP9 (A-kinase anchoring protein 9; AKAP450) (Takahashi et al., 2002; Ong et al., 2018), and pericentrin (Kendrin) (Zimmerman et al., 2004; Lawo et al., 2012) are also important for centrosomal localization of the complex, but since they are incorporated into PCM, they may also indirectly modulate γ-TuRC binding. Spatially and temporally distinct subpopulations of γ-TuRCs in centrosomes may be involved in different functions. In addition to canonical microtubule nucleation, γ-TuRC participates in centriole biogenesis and stabilization, and in microtubule anchoring (Schweizer and Lüders, 2021; Vineethakumari and Lüders, 2022). The structural elements of γ-TuRC and the major regulatory proteins of centrosomal microtubule nucleation in mammalian interphase cells are summarized in Table 1.

**TABLE 1:** Building components of γ-TuRC and major regulatory proteins of centrosomal microtubule nucleation in mammalian interphase cells.


**γ-TuRC**	γ-tubulin, GCP2, GCP3, GCP4, GCP5, GCP6, Actin, MZT1, MZT2
**Activating**	CDK5RAP2, NME7, chTOG/XMAP215,TPX2
**Targeting/Anchoring**	CDK5RAP2, NEDD1, AKAP9, Cep192, Ninein, Pericentrin
	Dynein complex, FSD1, MSD1-WDR8, Augmin complex

Phosphorylation of targeting/anchoring proteins affects recruitment of γ-TuRCs to centrosomes. NEDD1 is phosphorylated at multiple sites (Gomez-Ferreria et al., 2012), and sequential phosphorylation of NEDD1 by Cdk1 (cyclin-dependent kinase 1) and Plk1 (polo-like kinase 1) is essential for centrosomal targeting of γ-TuRC (Zhang et al., 2009). Phosphorylation of NEED1 by Cdk1 is required for its interaction with Plk1 and allows binding of γ-TuRC to pre-existing microtubules *via* the multiprotein augmin complex (Haren et al., 2009; Johmura et al., 2011). The kinase Aurora A phosphorylates NEDD1, which is a prerequisite for nucleation of microtubules from chromatin (Pinyol et al., 2013; Scrofani et al., 2015). Moreover, phosphorylation of NEDD1 by PLK4 promotes its interaction with SAS-6, the central component of the centriolar cartwheel (Chi et al., 2021), which associates with γ-TuRC during initiation of centriole duplication (Gupta et al., 2020). Phosphorylation of pericentrin by Plk1 (Santamaria et al., 2011) supports the accumulation of NEDD1 and CEP192 at the centrosome (Lee and Rhee, 2011). Phosphorylation of proteins participating in the recruitment of γ-TuRC to centrosomes therefore plays an important role in centriole biogenesis and microtubule nucleation.

Besides targeting/anchoring proteins, several modulatory proteins, not covered in this review, are also critical for regulating microtubule nucleation from centrosomes (Sulimenko et al., 2017). These proteins likely affect microtubule nucleation more indirectly. As an example, TACC3 (transforming acidic coiled-coil containing protein 3) stabilizes γ-TuRC during its assembly from γ-TuSC (Singh et al., 2014; Rajeev et al., 2019). On the other hand the putative tumor suppressor cyclin-dependent kinase five regulatory subunit-associated protein 3 (CDK5RAP3; C53) (Wang et al., 2007), which exerts multiple functions in cell cycle regulation, DNA damage response, cell invasion, and ER homeostasis (Sheng et al., 2021), interacts with γ-TuRC and acts as a negative regulator of microtubule nucleation. Displacement of C53 from the centrosome by exposure of cells to ER stress stimulates microtubule nucleation (Klebanovych et al., 2022). Intriquingly, some GTPase-activating proteins (GAPs) for ARF small GTPases (Sztul et al., 2019) may also be involved in regulating centrosomal microtubule nucleation. GAP ELMOD2, which acts with the GTPase ARL2, associates with centrosomes, and its deletion suppresses γ-TuRC recruitment and microtubule nucleation (Turn et al., 2020). Similarly, GAP GIT1, which acts with the GTPase Arf6 and functions as signalling adaptor protein, also associates with centrosomes (Zhao et al., 2005). Depletion of GIT1 suppresses centrosomal γ-tubulin accumulation and microtubule nucleation (Sulimenko et al., 2015; Černohorská et al., 2016). This suggests that signalling pathways other than those involving kinases and phosphatases may be involved in the regulation of γ-TuRC-dependent microtubule nucleation.

Interestingly, interactions between γ-tubulin and proteins essential for nonmuscle actin assembly, such as the Arp2/3 complex and its activator WASH, have been reported in different systems (Schaerer-Brodbeck and Riezman, 2003; Monfregola et al., 2010). Arp 2/3 requires profilin 1 for actin assembly, which sequesters actin, accelerates actin nucleotide exchange, and can dock to free actin filament (+)-ends as profilin-actin. Profilin 1 plays a key role in coordinating the different sub-arrangemeents in dynamic actin cytoarchitecture (Henty-Ridilla and Goode, 2015). Profilin 1 associates with γ-TuRC and its deletion enhances centrosomal microtubule nucleation in interphase cells (Nejedlá et al., 2021). As centrosomes have been proposed to nucleate actin polymerization (Farina et al., 2019; Inoue et al., 2019), it is possible that loss of profilin 1 results in less polymerization-ready actin (profilin-actin) and fewer actin filaments around centrosomes. The reduced steric hindrance could lead to increased *de novo* microtubule nucleation, as has been proposed for mitotic centrosomes (Plessner et al., 2019). Alternatively, deletion of profilin 1 would make more actin accessible for association with γ-TuRCs, which in turn would increase functional complexes formation and microtubule nucleation. The activity of γ-TuRC may therefore play an important role in centrosomal microfilament/microtubule cross-talk (Karlsson and Dráber, 2021).

### γ-Tubulin oligomers and filaments

Several studies using purified cellular or recombinant γ-tubulins have shown that γ-tubulin is capable of forming filamentous structures *in vitro*. The results of high-resolution microscopy suggest that such structures may also be present in cells, as documented below.

Acentrosomal plant cells contain large amounts of γ-tubulin compared to animal cells, and plant γ-tubulin forms heterogeneous complexes of high molecular weight (Dryková et al., 2003). Immunopurification of γ-tubulin with an anti-peptide antibody to γ-tubulin was performed from *Arabidopsis thaliana* cells. Analysis of the purified γ-tubulin with negative staining and transmission electron microscopy (TEM) revealed helically entangled double filaments together with filament bundles. Atomic force microscopy (AFM) showed that the most common width of the double-stranded filaments is 8.5 nm, which corresponds to the width inferred from TEM analysis (∼6 × 9 nm in a cross-section). When overexpressed GFP-labeled γ-tubulin was purified from *Arabidopsis* cells with anti-GFP antibody and acid elution, immunofluorescence microscopy revealed fibrillar structures. When purification was performed at a low SDS concentration that interfered with the interactions between γ-tubulin and GCPs, short γ-tubulin filaments were also detected. This suggests that *Arabidopsis* γ-tubulin is capable of forming filaments *in vitro* in the absence of GCPs (Chumová et al., 2018). Such formation of γ-tubulin filaments was not restricted to plant cells. When overexpressed RFP-labeled γ-tubulin from human osteosarcoma cells U2OS was purified using anti-RFP antibody and acid elution, immunofluorescence microscopy revealed filaments. In the absence of GCPs, filaments were also formed, but they were shorter. TEM confirmed the double-stranded character of the filaments (Chumová et al., 2018). Oligomerization of γ-tubulin has been previously reported in microtubule proteins isolated from porcine brain by two temperature-dependent cycles of polymerization and depolymerization (MTP-2). MTP-2 preparations electrophoretically separated under nondenaturing conditions generated “ladders” of multiple oligomers containing α-tubulin and γ-tubulin (Sulimenko et al., 2002). After isolation of γ-tubulin from MTP-2 with an anti-peptide antibody to γ-tubulin and immunizing peptide elution, γ-tubulin oligomers were detected in samples lacking αβ-tubulin dimers. Moreover, purified γ-tubulin from brain lacking both GCPs and αβ-tubulin dimers was capable of forming oligomers (Chumová et al., 2018).

Formation of γ-tubulin oligomers *in vitro* was also observed in the case of isolated recombinant proteins. TEM analysis of purified His_6_-labeled human γ-tubulin expressed in *E. coli* revealed a meshwork of γ-tubulin filaments termed γ-strings (Rosselló et al., 2018). Purified recombinant human γ-tubulin expressed in *E. coli* formed conformationally distinct aggregates, including long thin fibers ∼6.7 nm wide, in the presence of ATP and chaperonin CCT of type II (Pouchucq et al., 2018). Interestingly, purified Tev-StrepII-His6-labeled human γ-tubulin produced in Sf9 insect cells by a baculovirus expression system self-assembled into filaments with variable width at high γ-tubulin concentration (1–2 μM) (Thawani et al., 2020). 3D reconstructions of negatively stained electron micrographs of thin width γ-tubulin filaments revealed four linear arrays of interacting γ-tubulins. When the crystal structure of human γ-tubulin (PDB: 1Z5W) (Aldaz et al., 2005) was docked to the reconstituted filaments, a lateral arrangement of γ-tubulin in a linear array was revealed with a repeat unit of approximately 54 Å (Thawani et al., 2020). This closely matched the lateral repeats but not the longitudinal repeats (40 Å) of αβ-tubulin in microtubule lattice (PDB: 6DPU) (Zhang et al., 2018). Arrays of γ-tubulin were also generated from purified myc-His_6_-tagged human γ-tubulin expressed in Sf9 cells at concentrations of 0.25 μM and above. Helical reconstruction of negative-stain electron micrographs of the γ-tubulin arrays revealed a fivefold symmetry with a hollow center and a diameter of ∼15 nm. Docking of the crystal structure of γ-tubulin to 3D reconstruction of electron micrographs disclosed a lateral arrangement of γ-tubulins along the long axis with their (+) ends facing outward enabling interaction with αβ-tubulin. The γ-tubulin arrays promoted formation of microtubules and nucleation capacity correlated with array formation (King et al., 2020). Short templating γ-tubulin oligomers might enhance the rate of spontaneous αβ-tubulin assembly by eliminating kinetic barrier to lateral αβ-tubulin growth (Rice et al., 2021). Overall, the results of the *in vitro* experiments demonstrate the intrinsic capability of γ-tubulin to form oligomers and filaments.

γ-Tubulin arrays were detected *in vivo* in interphase cells. Association of γ-tubulin along pre-existing microtubules has been observed in higher plants (Liu et al., 1993) or fission yeasts (Sawin et al., 2004). In S2 cells of *Drosophila melanogaster*, γ-tubulin localized along interphase microtubules in the form of γ-TuRC, and it was proposed that the γ-TuRC could regulate microtubule dynamics by limiting catastrophes (Bouissou et al., 2009). γ-Tubulin was found on microtubules forming a marginal band in erythroid cells of the chicken embryo (Linhartová et al., 2002), and in cultured mammalian cell lines in interphase, where it sporadically coated microtubules in limited regions (Hubert et al., 2011). In contrast, tubular γ-tubulin structures that were not associated with microtubules were found in the fraction of cells overexpressing tagged γ-tubulin, suggesting that γ-tubulin retains the potential to assemble into macromolecular assemblies *in vivo* (Shu and Joshi, 1995). Interestingly, superresolution microscopy in *Arabidopsis* cells revealed short γ-tubulin filaments outside the microtubules. They accumulated both at the mitotic spindle poles and at the outer membrane of the nuclear envelope. It has been suggested that γ-tubulin may form a dynamic 3D structure of more or less densely packed, laterally connected filaments (Chumová et al., 2018). Such fibrillar structures were distinct from the dynamic polar fibers termed γ-tubules that have been detected in mammalian tissue culture cell lines and reportedly to be formed in a GTP-dependent manner from γ-TuRCs and pericentrin (Lindström and Alvarado-Kristensson, 2018). However, pericentrin is not present in the *Arabidopsis* genome. The role of fibrillar γ-tubulin assemblies is currently unclear. It has been proposed that they have sequestration and scaffolding functions (Chumová et al., 2021). They may also participate in mechanotransduction processes, as they are associated with the multiprotein complex LINC (linker of nucleoskeleton and cytoskeleton) (Rosselló et al., 2018; Chumová et al., 2019; Corvaisier and Alvarado-Kristensson, 2020). Interestingly, γ-tubulin has been detected in inner membranes and matrix of isolated mitochondria (Dráberová et al., 2017), and it has been suggested that γ-tubulin filaments (γ-strings) may represent mitochondrial structural components (Lindström et al., 2018). As described in the following section, γ-tubulin can also be found in cell nuclei. It has been proposed that γ-tubulin filaments may also play a structural role in nuclei (Corvaisier et al., 2021). Further studies are however needed to verify the presence of fibrillar γ-tubulin assemblies in different model systems, determine their composition, structure and decipher their cellular function(s).

### γ-Tubulin nuclear functions

Contrary to the persistent view that γ-tubulin is a typical cytosolic protein, γ-tubulin has been localized in the nuclei of both plant (Binarová et al., 2000) and animal cells (Lesca et al., 2005; Höög et al., 2011). In addition, specific nuclear localization signal (NLS) in the γ-tubulin molecule was deciphered (Höög et al., 2011). Proteomic analysis suggested that γ-tubulin might be also in nucleoli (Andersen et al., 2002). A significant increase in γ-tubulin protein level observed in glioblastoma cell lines (Katsetos et al., 2009) contributed to the unequivocal confirmation of nucleolar γ-tubulin (Hořejší et al., 2012). Surprisingly, GCP2 and GCP3 were also found in nucleoli, although no NLSs were identified in these molecules. This suggests that both proteins might enter the nucleus by hitchhiking on γ-tubulin (Dráberová et al., 2015).

There is evidence that γ-tubulin has nuclear-specific functions. It has been reported that BRSK1-mediated phosphorylation of γ-tubulin at S385 leads to transient nuclear accumulation of γ-tubulin in S phase of cell cycle (Eklund et al., 2014). Nuclear γ-tubulin attenuates the activity of E2F transcription factors, important regulators of cell cycle progression, in both animals (Höög et al., 2011) and plants (Kállai et al., 2020). It was found that γ-tubulin and DP1 (E2F heterodimerization protein) compete for the same binding site on E2F and that the tumor suppressor retinoblastoma protein 1 (RB1) and γ-tubulin regulate each other’s expression. Interestingly, a proapoptotic effect was observed in cancer cells with nonfunctional RB1 signaling after depletion of γ-tubulin protein levels (Ehlén et al., 2012). The E2Fs-γ-tubulin interactions may participate in coordinating genome duplication with spindle assembly in both animal cells containing centrosomes and in acentrosomal plant cells in which microtubules are nucleated from dispersed sites (Binarová et al., 2006; Pastuglia et al., 2006). In addition, E2Fa and RBR1 (*Arabidopsis* homolog of RB1) form foci in plant cells in response to double-strand breaks that seem to allow recruitment of the repair protein Rad51 (Biedermann et al., 2017; Horvath et al., 2017). In mammalian cells, Rad51 interacts with γ-tubulin in response to DNA damage (Lesca et al., 2005). These results suggest that E2Fs-γ-tubulin complexes may promote DNA repair or control the expression of genes related to DNA repair (Raynaud and Nisa, 2020).

γ-Tubulin colocalizes in nucleoli with a putative tumor suppressor C53, and it has been shown that C53 inhibits G_2_/M checkpoint activation by DNA damage. Overexpression of γ-tubulin counteracts this C53 action (Hořejší et al., 2012). Besides, γ-tubulin may be involved in DNA damage repair processes as it associates not only with Rad51 (Lesca et al., 2005) but also with BRCA1 (Hubert et al., 2011), and ATR (Zhang et al., 2007). Proliferating cell nuclear antigen (PCNA) is a coordinator of DNA replication and repair (Stoimenov and Helleday, 2009). It has been reported that γ-tubulin binds PCNA and aids in its recruitment to chromatin in mammalian cells. A positive correlation between γ-tubulin and PCNA expression was found in all examined tumor types (Corvaisier et al., 2021). Finally, γ-tubulin is capable to modulate the anaphase-promoting complex/cyclosome (APC/C), which is a large protein complex with multiple subunits that is important for cell cycle regulation. There is strong evidence that in *Aspergillus nidulans* γ-tubulin plays an important role in regulating APC/C during interphase (Nayak et al., 2010) by inactivation of the APC/C activator CdhA (*A. nidulans* homolog of Cdh1) at the G1-to-S transition (Edgerton-Morgan and Oakley, 2012). Deciphering the molecular mechanisms underlying the various nucleus-specific functions of γ-tubulin remains the major challenge for future studies.

## Concluding remarks

Recent structural studies of γ-TuRCs have been very informative, but molecular mechanisms how factors involved in promoting the transition from the open to the closed state of γ-TuRCs needs to be thoroughly characterized. Many proteins (targeting, activating, anchoring, modulating) that interact with γ-TuRCs are required to nucleate microtubule at right place and time. However, the upstream signaling pathways ensuring that these regulatory proteins act in concert and initiate microtubule nucleation according to the cell’s requirements are largely unknown. It is becoming increasingly clear that kinases and phosphatases are important for microtubule regulation. Therefore, functional characterization of phosphorylation sites in γ-TuRCs and interacting proteins is required. Another important issue to be resolved is the analysis of γ-TuRC subpopulations that differ in composition or PTMs. Future studies are also needed to determine whether different γ-TuRCs can independently nucleate cell type-specific noncentrosomal microtubules. A detailed understanding of the molecular mechanisms of microtubule nucleation should provide new insights into the importance of γ-TuRC dysregulation in cancer cell behaviour and in neurological diseases and could lead to the development of highly specific γ-tubulin drugs (Dráber and Dráberová, 2021).

In recent years, the functions of γ-tubulin independent of microtubule nucleation have received more attention. High-resolution cryo-electron microscopy will be essential for deciphering the structure of recently reported γ-tubulin fibers and their high-level assemblies in a cellular context. Understanding the role of γ-tubulin isotypes under different stress conditions, in cell cycle checkpoints and in DNA repair will be important to elucidate their roles in carcinogenesis.

Finally, it has become increasingly evident that microtubules and microfilaments frequently cooperate. Recent work suggests that both microtubules and actin filaments are nucleated from centrosomes and that actin and its associated proteins control microtubule nucleation. Sophisticated *in vitro* reconstitution experiments should shed light on the role of proteins regulating microtubule nucleation in the cross-talk between microtubules and microfilaments.
